# The impact of cardiomotor rehabilitation on endothelial function in elderly patients with chronic heart failure

**DOI:** 10.1186/s12872-021-02327-5

**Published:** 2021-11-01

**Authors:** Juming Chen, Shenhong Gu, Yanling Song, Xinbo Ji, Wangyuan Zeng, Xiaoxi Wang, Yachun Wang, Qingfeng Feng

**Affiliations:** 1 Department of General Practice, The First Affiliated Hospital of Hainan Medical University, Haikou City, 570102 Hainan Province China; 2 Graduate School, Hainan Medical University, China Medical University, Haikou City, 571199 Hainan Province China

**Keywords:** Heart failure, Chronic heart failure, Exercise rehabilitation, Endothelial progenitor cells, PI3K/AKT

## Abstract

**Background:**

To explore the effects of cardiac exercise rehabilitation on peripheral blood endothelial progenitor cells (EPC) in elderly patients with chronic heart failure.

**Methods:**

80 elderly patients with chronic heart failure were selected from March 2017 to March 2019 and randomly divided into two groups (N = 40). The control group was treated routinely and walked freely for 30–60 min every day. The patients in the exercise rehabilitation group developed a cardiac exercise rehabilitation plan. Then, cardiac function and peripheral blood B-natriuretic peptide (BNP) levels in the two groups were compared. The cell viability, proliferation, apoptosis, and invasion ability of EPCs were detected. The levels of the PI3K/AKT pathway and eNOS and VEGF were compared.

**Results:**

There were no significant differences in all indexes between the two groups before treatment (*P* > 0.05), and both improved significantly after treatment (*P* < 0.05). After treatment, LVEF and LVFS in the exercise rehabilitation group were significantly higher than those in the control group (*P* < 0.05), and LVEDD and LVESD were significantly lower than those in the control group (*P* < 0.05). The BNP level in the exercise rehabilitation group was significantly lower than that in the control group (*P* < 0.05). The cell viability, proliferation, invasion ability of EPC, and the levels of PI3K, AKT, eNOS, and VEGF mRNA and protein in the exercise rehabilitation group were significantly higher than those in the control group. Apoptosis rate was significantly lower than those in the control group (*P* < 0.05).

**Conclusions:**

Visceral exercise rehabilitation can improve cardiac ejection and myocardial function in elderly patients with chronic heart failure, and can promote the vitality, proliferation, and invasion of peripheral blood EPC, and promote the expression of eNOS and VEGF by upregulating the PI3K/AKT pathway to promote angiogenesis and endothelial function.

**Supplementary Information:**

The online version contains supplementary material available at 10.1186/s12872-021-02327-5.

## Background

The incidence rate of heart failure (HF) is increasing year by year. In developed countries, the prevalence of chronic heart failure (CHF) is over 10% in the population over 70 years old [1]. Although the incidence rate of new CHF has dropped significantly in the past decades, the number of hospitalized patients, arrhythmia, and quality of life of patients has not improved significantly [2]. The treatment of CHF in the advanced stage is limited. There are many limitations in the treatment of heart transplantation and left ventricular-related devices. CHF cannot be cured and often requires long-term treatment and follow-up visits. A large number of clinical studies show that cardiac exercise rehabilitation can significantly improve the exercise tolerance of patients with HF and improve the cardiac function, quality of life, mortality, and prognosis of patients [3].

The main manifestation of HF is the decrease of cardiac ejection function and myocardial damage that causes the upregulation of B-type brain natriuretic peptide (BNP) in peripheral blood. The research shows that the damage of blood vessel structure is one of the mechanisms that affect heart function. The damage of microvascular in the heart tissue will cause blood circulation disorder, apoptosis, and damage of cardiac myocytes. Then, it affects the ejection fraction of the heart, increases the level of BNP in serum. Endothelial progenitor cells (EPC) play an important role in the treatment of HF. The decrease of EPC level in peripheral blood has a certain relationship with the poor prognosis of patients with HF [4]. EPC, as the precursor of endothelial cells, has the characteristics of migration and differentiation. It can further proliferate and differentiate into mature endothelial cells when blood vessels are injured. EPC from bone marrow can migrate to peripheral blood and participate in the repair and angiogenesis of damaged blood vessels under the stimulation of physiological or pathological factors [5].

Based on studies, it has been observed that EPC dysfunction occurs in patients with CHF [6, 7]. Multiple approaches were made in various studies in order to improve EPC dysfunction in patients with HF. According to the literature, epigenetic mechanisms also play a role in the repair process, such as DNA methylation [8]. A study by Huang WP et al. suggested that Fenofibrate reverses EPC dysfunction [9]. Another study by Jung C et al. suggested that eplerenone positively affects EPC dysfunction in patients with CHF [10]. A recent study suggested that cardiac resynchronization therapy (CRT) can improve circulating EPCs in patients with HF [11]. A prior report suggested that exercise training help improve endothelial function [12]. Nonetheless, along with other investigators, the European Society of Cardiology (ESC) guidelines also recommended regular aerobic exercise for improving functional capacities and symptoms [13].

PI3K/AKT pathway plays an important role in the proliferation and function of EPC. The activation of the PI3K/AKT pathway can improve the cell viability of EPC and promote the invasion ability and repair function of EPC. On the other hand, the activation of the PI3K/AKT pathway can also promote eNOS and VEGF, which can improve endothelial function [14]. The study shows that activation of the PI3K/AKT signaling pathway can inhibit the process of myocardial fibrosis and improve heart function, reduce the degree of HF.

This study has mainly analyzed the therapeutic effects of cardiac exercise rehabilitation on elderly patients with CHF and the influence of EPC function and PI3K/Akt pathway in peripheral blood. It preliminarily explored the therapeutic mechanisms of cardiac exercise rehabilitation and to see whether the outcome can contribute to the cardiac ejection capacity and myocardial functions in patients with CHF.

## Methods

### Study design

This is an interventional study that analyzed the therapeutic mechanisms of cardiac exercise rehabilitation on CHF patients. The study included 80 elderly patients with CHF from March 2017 to March 2019 as the study subjects. The patient's ages were between 65 and 77 years, and 46 males and 34 females. The patients were randomly divided irrespective of gender into the control and exercise rehabilitation groups, with 40 cases in each group.

### Ethical approval and consent

This study has been approved by the ethics committee of the First Affiliated Hospital of Hainan Medical College, and all the experimental protocol for involving humans was in accordance with the guidelines of the Declaration of Helsinki. Informed consent was obtained from each patient before the implementation process of this study.

### Inclusion and exclusion criteria

Patients aged between 65 and 80 who met the diagnostic criteria for heart failure [7] and classified as heart failure of grade II or III with exercise ability were included in this study.

Correspondingly, patients classified as heart failure of grade III or above who were unable to exercise; patients with severe emphysema, asthma, and other diseases such as lower extremity movement disorder and disability that could affect free exercise were excluded from this study. Patients who could not complete daily exercise tasks regularly were also excluded from this study.

### Intervention methods

According to the guidelines published by Xuezhai et al., both the control group and exercise rehabilitation group in our study received conventional treatment based on the specific conditions of each patient [15]. These conventional treatments included primary disease control, diuresis, vasodilation, salt restriction, and the drugs used in these patients were based on the individual conditions of the patients. These drugs were β-blocker (metoprolol; Er, H32025391, AstraZeneca, 12–25 mg twice daily), digitalis (H33021566, Zhejiang Jinhua Kangenbei, 0.05–0.1 mg once daily), captopril (H44020939, a special medicine Industry, 25 mg twice daily).

In addition to the conventional treatment above, the control group only performed simple exercises at the bedside or indoors, while the exercise rehabilitation group performed rehabilitation exercises for 12 weeks and walked freely for 30–60 min a day. The exercise group performed cardiac exercise rehabilitation: 12 weeks, 3–5 times a week. The exercise prescription is based on the simplified three-stage exercise rehabilitation program of HF, combined with the classification of patients' heart function, the condition of patients' activity ability and metabolic equivalents (MetS), and walking length to the converse. Metabolic equivalent (MetS) = {[velocity (m/min) × 0.1 + [slope(decimal) × velocity(m/min) × 1.8] + 3.5}/3.5

Exercise rehabilitation program for a patient with HF of cardiac function level III [16, 17]: First, bedside rehabilitation is performed: stand beside the bed → move → support walking exercise, with 350–700 m each time, 3–4 times/day; when patients feel that they can bear such training intensity, and can carry out indoor rehabilitation. Indoor rehabilitation: patients need to repeat bedside bed walking → with 100–250 m/time as a warm-up before training, and then instruct patients to walk 350–1050 m every day, 1–2 times a day Three months as a course of treatment;

Exercise rehabilitation program for heart failure patients with level II cardiac function [16, 17]: First, the patient needs to walk 250–500 m/time in the corridor, and go upstairs with help, 1–2 times/day. If the patients can still bear the indoor walking, they will walk outside, first, walk 500–1000 m as a warm-up, and then ask the patients to walk 1400–2800 m/day, 1–2 times/day. Patients need to exercise according to the above guidelines.

### Left ventricular angiography

The left ventricular angiography was used to detect the cardiac function indicators. The pigtail catheter was inserted retrogradely into the left ventricle from the femoral artery or brachial artery through a percutaneous puncture. The right cardiac catheter entered the left ventricle through the patent foramen ovale or abnormal intracardiac channel. According to the operating standards, a 4F catheter (TERUMO salary, Japan) was placed in the left ventricle, and the matching contrast agent was injected. The dose was 35 mL, the flow rate was 15–20 mL/s, and then the contrast-detection was performed. The contrast position is positive or lateral or oblique or axial. Testing items include: left ventricular ejection fraction (LVEF), left ventricular short-axis shortening rate (LVFS), left ventricular end-diastolic diameter (LVEDD), left ventricular end-systolic diameter (LVESD). After the test, the catheter was quickly pulled out, and the local compression was followed by compression bandaging.

### ELISA

5 ml of peripheral venous blood was taken before and after the treatment. The upper serum was centrifuged (2000 rpm, 10 min) after standing. The BNP level was detected by enzyme-linked immunosorbent assay (ELISA), while the range of normal BNP is 0–100 pg/mL. The reagent was added according to the kit's instructions (Beyotime, Shanghai, China). Then the Imark microplate reader (Bio-Rad, California, USA) was used to calculate the concentration according to the standard curve.

### Collection of EPC

10 ml of venous blood of two groups of patients were extracted before and after treatment. Based on the methodological approach by Nan et al. [18], peripheral blood EPC were separated by density gradient centrifugation using the human peripheral blood endothelial progenitor cell isolation kit (Haoyang Bioproducts Technology Co., Ltd., China). The EPC was cultured and grown in an EGM-2 Bullet Kit medium (Lonza) and collected. The 8th to 12th passage cells were subjected to follow-up experiments. Then, the cells were labeled by Dil-ac-LDL and FITC-UEA-I reagent (Becton, Dickinson and Company, New Jersey, USA). The proportion of double-positive cells was 97.43% detected by flow cytometry.

### CCK-8 detection of cell growth

2 × 10^4^ cells were inoculated into 96 well plates, and 10 μl CCK-8 reagent (Beyotime, Shanghai, China) was added to the plate 48 h after culture. The cells were cultured at 37 °C, and the relative cell activity was calculated by microplate reader at an optical density of 450 nm.

### Clonogenesis experiment

According to the method of reference, 1 × 10^4^ cells were inoculated into a 6-well plate and cultured in a 5% CO_2_ incubator at 37 °C for 2 weeks. After the medium was sucked out, 500 μl methanol solution was added to each hole to fix the cells for 15 min. Then 1 ml crystal violet dye (Beyotime, Shanghai, China) was added. The solution was incubated for 20 min and counted the number of clone formations.

### Flow cytometry

Apoptosis rate was measured by flow cytometry, and 1 × 10^6^ cells were digested by 0.25% trypsin without EDTA. Cells were washed with 5% BSA, centrifuged at 2000 rpm for 5 min, and resuspended in PBS. Then 5 μl annexin (20 μg/ml, sigma company, USA) was added and incubated in the dark for 15 min. Then 10 μl propidium iodide (PI, 50 μg/ml, sigma company, USA) was incubated in the dark for 15 min. The apoptosis rate was detected by FACSCanto II flow cytometry (Becton, Dickinson and Company, New Jersey, USA).

### Transwell assay for cell invasion

Matrix gum (BD Biosciences, New Jersey, USA) was added in the upper chamber of the Transwell cell (Invitrogen, CA, USA). A complete medium (Invitrogen, Carlsbad, CA, USA) was added to the bottom chamber. Then 3 × 10^4^ cells were added to the upper chamber for 48 h. After 48 h, the cells that did not invade the basement were washed away. Then the cells were fixed with 20% methanol and stained with 0.2% crystal violet. Under the inverted microscope, the number of cells invading the bottom chamber of each field was counted.

### RT-qPCR

The mRNA expression levels of PI3K, AkT, eNOS, and VEGF in two groups of EPC were detected by RT-qPCR. The cells were grinded and lysed under the protection of liquid nitrogen. Then the total RNA was extracted and dissolved in DEPC water. The reverse transcription kit (Roche, Basel, Switzerland) synthesized cDNA under 37 °C/15 min and 85 °C/15 s conditions. SYBR Green PCR Master Mix qPCR kit was used to carry out qPCR experiment, DNA polymerase was activated at 95 °C/5 min. Two-step PCR (95 °C/10 s and 60 °C/30 s) was carried out in 40 cycles, extending at 75 °C/10 min, and finally maintaining at 4 °C. GAPDH was used as an internal parameter to compare the circulating threshold (ΔΔCT) and analyze the expression level of RNA. The mRNA sequences of PI3K, AKT, eNOS, and VEGF were retrieved from the PubMed database. Primers were designed and synthesized by TaKaRa Biotechnology (Dalian, China) company. The primer sequences are as follows:

**Table:** 

Genes	Forward	Reverse
PI3K	CCAAGAGGGTACAGCAAAGAAT	TGGGTGGTCCAGGGTTTCTT
AKT	TGGGTGGTCCAGGGTTTCTT	CTGCAGGGGGGTGATATGT
eNOS	GGCGTCTTCAGAGCTGTACAC	CTAAGGCGGTTGGCACTTCATA
VEGF	GCAATGATGAAGCCCTGGAGT	TTCTCCGCTCTGAACAAGGCT
GAPDH	CCTGAAGTACCCCATTGAACAC	CTCATTGCCGATAGTCATGACC

### Western blot

The expression levels of PI3K, AKT, eNOS, and VEGF were detected by Western blot. Cells were grinded in liquid nitrogen and washed with precooled PBS buffer. Then, the samples were split in cold Ripa cracking buffer (Beyotime, Shanghai, China). The total protein content was measured by the BCA kit (Aapplygen, Beijing, China). Then the total protein was separated by 10% gel SDS-PAGE (120 V electrophoresis 90 min). The protein was transferred to the PVDF membrane (Bio-Rad, California, USA) (50 V, 120 min). It was sealed in a closed solution containing 5% skim milk. Then, PDVF membrane was combined with anti-PI3K (ab154598, Abcam, Cambridge, USA), anti-AKT (ab8805, Abcam, Cambridge, USA), anti-eNOS (ab76198, Abcam, Cambridge, USA), and anti-VEGF (Abcam, Cambridge, Ma, USA) (1:1000 dilution) were incubated overnight at 4 °C and then, incubated at room temperature for 1 h with 1:5000 diluted HRP coupling second antibody. The protein bands were visualized by the ECL kit. All antibodies were purchased from Abcam (USA). GAPDH was used as the internal parameter, and the gray level of protein bands was detected by imagePD software.

### Statistical analysis

In this study, SPSS 19.0 was used for data analysis. The χ^2^ test was used for clinical count data (%), and the t-test was used to compare two groups of measurement data ($\overline{x} \pm s$). Three multiple holes were set up in all the experiments in the laboratory. Statistically significant is *P* < 0.05.

## Results

### Characteristics of the study population

In this study, 80 elderly patients with chronic heart failure were included and randomly divided into two groups (n = 40/each group). Among the total subjects, 46 were males, and 34 were females. There was no significant difference between the two groups in general data, which was comparable (*P* > 0.05) (see Table 1). Patients with other co-morbidities such as diabetes mellitus were managed accordingly so that the results were not influenced by EPCs function.

**Table 1:** Comparison of basic information between the two groups

Group	N	Age	Gender		Cardiac function Grades		LVEF (%)	LVFS (%)	LVEDD (mm)	LVESD (mm)
			Male	Female	II	III				
Control group	40	69.28 ± 2.34	22	18	27	13	43.41 ± 3.28	15.44 ± 3.15	57.38 ± 4.21	48.53 ± 4.24
Exercise rehabilitation group	40	70.04 ± 2.15	24	16	25	15	44.73 ± 3.02	15.16 ± 3.08	56.85 ± 3.98	47.38 ± 4.33
t/χ^2^		0.384	0.521		0.457		0.684	0.318	0.432	0.384
*P*		0.628	0.448		0.498		0.433	0.795	0.682	0.725

### Comparison of cardiac function indexes between the two groups

**Fig. 1:**
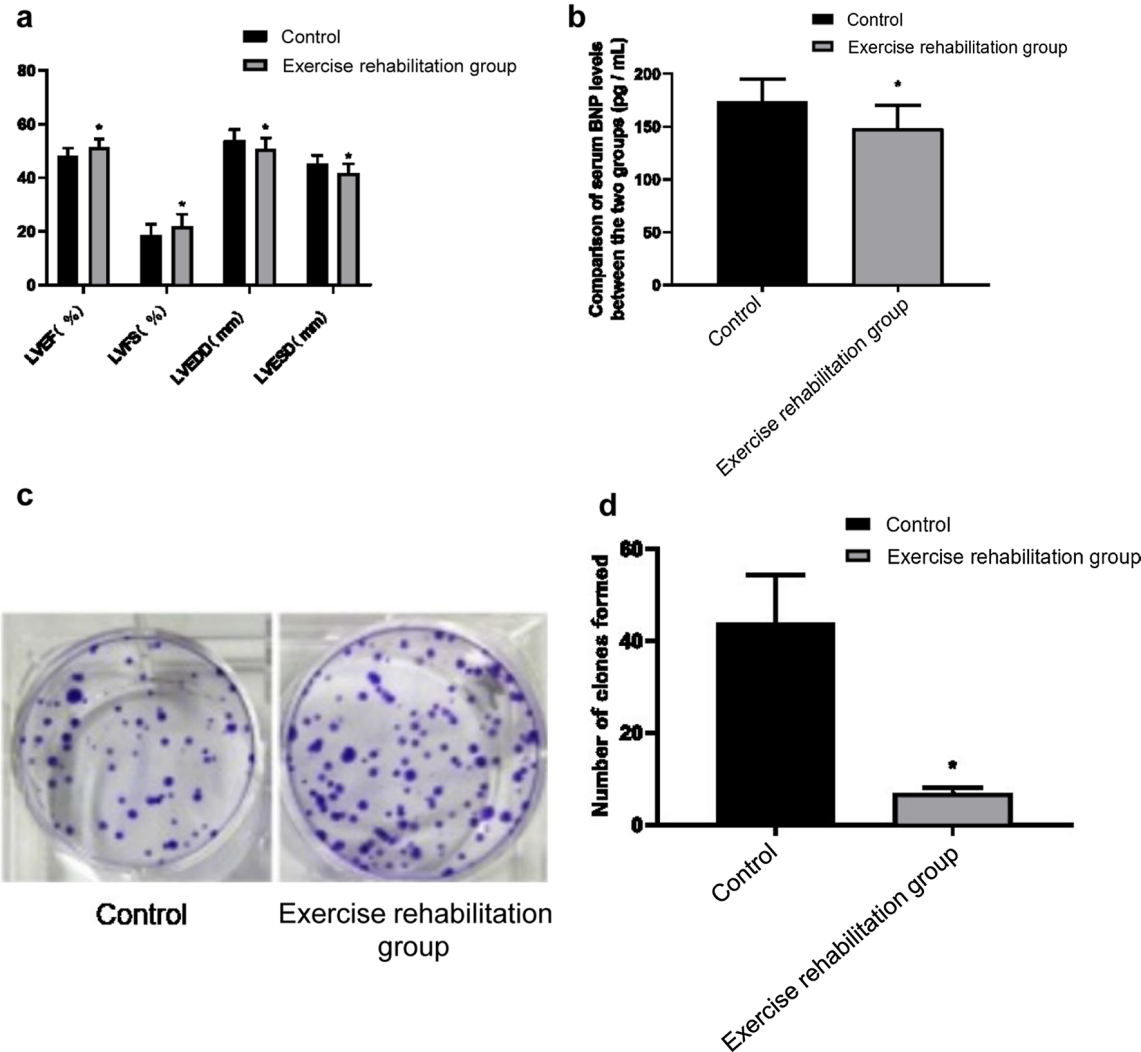
**a** The heart function index was detected by left ventriculography. A 4f catheter was used according to the operation standard. The detection items included: left ventricular ejection fraction (LVEF), left ventricular short-axis shortening rate (LVFS), left ventricular end-diastolic diameter (LVEDD), left ventricular end-systolic diameter (LVESD). **b** The BNP level in serum was detected by ELISA, 5 ml of peripheral venous blood was extracted before and after treatment, respectively, and the BNP level was detected by enzyme-linked immunosorbent assay. **c** 1 × 104 cells were inoculated into a 6-well plate and cultured in a 5% CO_2_ incubator at 37 °C for 2 weeks. Crystal violet dye was added, and the number of clones was counted. **d**. The statistical analysis of clone number (*P < 0.05)

After treatment, the indexes of heart function in the two groups were significantly improved (*P* < 0.05). After treatment, LVEF (51.27 ± 3.26%) and LVFS (21.89 ± 4.54%) in the exercise rehabilitation group were significantly higher than those in the control group (47.97 ± 3.14%, 18.53 ± 4.07%) (*P* < 0.05). LVEDD (50.75 ± 4.16 mm) and LVESD (41.69 ± 3.53 mm) were significantly lower than those in the control group (53.85 ± 4.19 mm, 45.12 ± 3.27 mm) (*P* < 0.05) (see Fig. 1a; Additional file 1: Table S1). This suggests that the rehabilitation of cardiac exercise is helpful to improve the cardiac function of elderly patients with chronic heart failure.

### Comparison of serum BNP levels between the two groups

There was no significant difference between the two groups before treatment (206.36 ± 24.68 pg/ml vs. 211.72 ± 24.08 pg/ml) (*P* > 0.05). After treatment, the BNP level of the control group and the exercise rehabilitation group decreased to 174.28 ± 20.86 pg/ml and 148.58 ± 21.82 pg/ml, respectively (*P* < 0.05). The BNP level of the exercise rehabilitation group was significantly lower than that of the control group (*P* < 0.05). (See Fig. 1b; Additional file 1: Table S2). This suggests that cardiac exercise rehabilitation can reduce the BNP level in serum of elderly patients with chronic heart failure and protect the damaged myocardium.

### Comparison of EPC cell viability and cell proliferation in each group

**Fig. 2:**
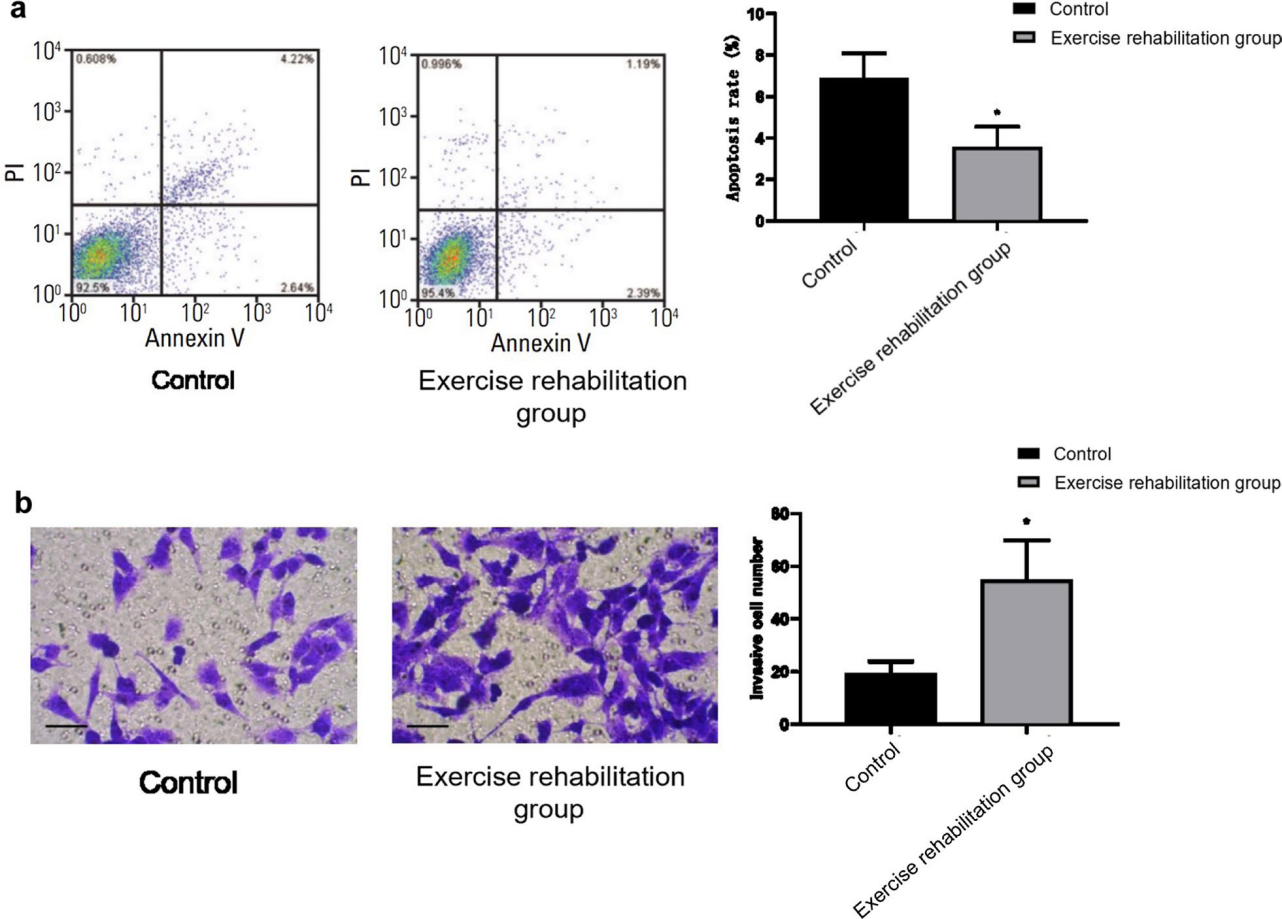
**a** Flow cytometry was used to detect the apoptosis effects of the control group and rehabilitation group. **b** The invasion ability of EPC was detected by the Transwell method

The relative cell viability 100.00 ± 3.98%, the number of clone formation 43.75 ± 10.54, and the apoptosis rate was 6.86 ± 1.24% of the control group. The relative cell viability of 119.63 ± 4.38%, the number of clone formation 102.57 ± 21.69, and the apoptosis rate 3.58 ± 0.97% of the exercise rehabilitation group were shown in Figs. 1c, d, and 2a. The EPC cell viability and cell proliferation ability of the exercise rehabilitation group were significantly higher than that in the control group. The apoptosis rate was significantly lower than that in the control group (*P* < 0.05) (Figs. 1c, d, 2a). This suggests that exercise rehabilitation can improve the cell viability and proliferation of EPC in elderly patients with chronic heart failure and inhibit the apoptosis of EPC.

### Comparison of invasion ability of EPC cells in each group

The invasion ability of EPC plays an important role in the migration and migration invasion to damaged blood vessels. Transwell was used to detect the impact of exercise rehabilitation on the invasion ability of EPC. The results showed that the number of invasive cells in the control group was 19.57 ± 4.36. The exercise rehabilitation group was 54.85 ± 14.97. The invasiveness number of EPC cells in the exercise rehabilitation group was significantly higher than that in the control group (*P* < 0.05), as shown in Fig. 2b. It is suggested that exercise rehabilitation can improve the invasive ability of EPC in elderly patients with chronic heart failure.

### Comparison of PI3K, AKT, eNOS, and VEGF mRNA in each group

**Fig. 3:**
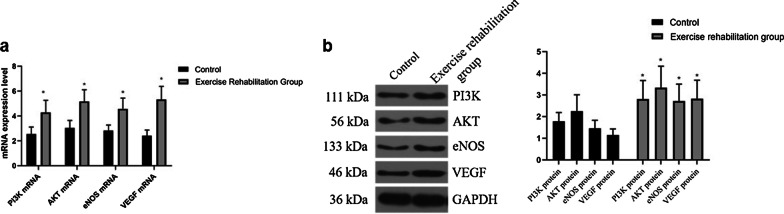
**a** qPCR was used to detect the mRNA expression of PI3K, AKT, eNOS, and VEGF of peripheral blood. **b** The expression level of PI3K, AKT, eNOS, VEGF, and GAPDH in the peripheral blood of each group was detected by western blotting

The levels of PI3K, AKT, eNOS, and VEGF mRNA in the exercise rehabilitation group were significantly higher than those in the control group (*P* < 0.05) (see Fig. 3a; Additional file 1: Table S3). It is suggested that exercise rehabilitation can improve PI3K, Akt, eNOS, and VEGF mRNA in EPC of elderly patients with chronic heart failure.

### Comparison of PI3K, Akt, eNOS, and VEGF proteins in each group

The protein levels of PI3K, AKT, eNOS, and VEGF in the exercise rehabilitation group were significantly higher than those in the control group (*P* < 0.05), as shown in Table2 and Fig. 3b. It is suggested that exercise rehabilitation can improve PI3K, AKT, eNOS, and VEGF in EPC of elderly patients with chronic heart failure.

**Table 2:** Comparison of PI3K, Akt, eNOS, and VEGF proteins in each group

Group	PI3K	AKT	eNOS	VEGF
Control group	1.78 ± 0.41	2.25 ± 0.76	1.46 ± 0.37	1.15 ± 0.28
Exercise rehabilitation group	2.81 ± 0.86	3.34 ± 0.99	2.72 ± 0.78	2.83 ± 0.85
t	10.815	9.114	18.254	21.841
*P*	0.000	0.000	0.000	0.000

## Discussion

This study asked whether cardiomotor rehabilitation can ameliorate endothelial function in elderly patients with chronic heart failure. The results showed that there was no significant difference between the two groups before treatment. There was an improvement in different degrees after treatment. After treatment, LVEF and LVFS in the exercise rehabilitation group were significantly higher than those in the control group. LVEDD and LVESD were significantly lower than those in the control group. In addition, there was no significant difference in serum BNP level between the two groups before treatment. After treatment, the BNP level of the control group and the exercise rehabilitation group decreased, respectively. The BNP level of the exercise rehabilitation group was significantly lower than that of the control group. In addition, the activity, proliferation ability, migration ability, PI3K, AKT, eNOS, and VEGF mRNA and protein levels of EPC in the exercise rehabilitation group were significantly higher than those in the control group. At the same time, the apoptosis rate was lower than that in the control group.

Exercise rehabilitation intervention can help to improve the treatment effects of chronic heart failure. In recent years, many clinical studies have confirmed that exercise rehabilitation intervention can help improve the treatment effects of chronic heart failure [19]. It is found that exercise rehabilitation training can effectively improve the heart function of patients with chronic heart failure. In this study, we simplified the three-stage rehabilitation program of heart failure, combined with the classification of heart function and activity ability of patients. We carried out a personalized, step-by-step rehabilitation strategy for each patient. BNP mainly exists in cardiomyocytes. When left ventricular heart failure occurs, BNP will be released to peripheral blood rapidly, which has a high diagnostic effect on heart failure. Moreover, the BNP level is a sensitive response index of myocardial function [20]. The results of this study showed that cardiac exercise rehabilitation could effectively reverse remodeling of cardiac structure and therefore bring functional improvement.

In order to further explore the mechanism of cardiac exercise rehabilitation to improve cardiac function, the biological behaviors of EPC in the two groups were tested and compared. EPC-induced angiogenesis and neovascularization are essential processes to improve cardiac function in heart ischemia-related diseases. EPC increased significantly in the early stage of HF, promoted angiogenesis, and protected endothelial cell function from restoring myocardial blood supply and improving myocardial function. At the same time, EPC decreased significantly in the late stage of heart failure [21]. Anti-heart failure treatments can improve the number of EPC in peripheral blood of patients with heart failure, promote the proliferation and migration of EPCs. In the EPC with cardioprotective effects, "late" EPC has a strong proliferation potential and has the ability to invade and integrate into damaged tissues and promote blood vessels [22]. The results of this study suggest that exercise rehabilitation can promote the activity, proliferation, and migration of EPC cells in the peripheral blood of an elderly patient with CHF to promote the repair function of EPC and improve cardiac function.

PI3K/AKT pathway is an important pathway regulating EPC proliferation, apoptosis, and invasion. In addition, activation of the PI3K/AKT pathway can also promote EPC cells to secrete NO and VEGF [23]. NO is a potent vasodilator, and its dynamic balance plays an important role in regulating vascular function. VEGF can promote angiogenesis and induce EPC to differentiate into endothelial cells to improve myocardial blood supply and improve myocardial function [24, 25]. Previous studies have shown that PI3K/AKT expression in rats with heart failure can be effectively promoted by cardiac shock wave therapy [26]. Zhao et al. [27] showed that activation of the PI3K/AKT pathway could promote the proliferation and angiogenesis of EPC cells. PI3K/AKT pathway can also improve EPC migration and angiogenesis by promoting the expression of MMP1 [14]. In addition, the PI3K/AKT pathway can reduce EPC dysfunction induced by high glucose by promoting the expression of eNOS [28]. In addition, some studies also show that exercise can promote the activation of the PI3K/ AKT pathway in cells and improve the function of cardiomyocytes or vascular endothelial cells [29, 30]. This suggests that cardiac exercise rehabilitation can improve PI3K/AKT pathway in EPC, which is the main mechanism to promote EPC proliferation and invasion and maintain EPC function.

This study has several limitations: (1) the present study only explored the impact of combined cardiomotor rehabilitation on cardiac function. (2) In this study, flow cytometry experiments were performed. However, reporting flow cytometry summary example along with FMO control explanation was not done. Therefore, we plan to use flow cytometry and report its summary example along with FMO control explanation in our future study on this topic. (3) This is preliminary research on this topic, and risk analysis is temporarily impossible to complete with current capabilities. Therefore, we plan to perform risk analysis in future experiments. (4) The study used a small sample size, and the results are preliminary. However, this study is a minuscule of a larger sample. Thus, an enlarged sample population and refined grouping strategy remain to be used further to confirm the results and conclusions of the present study.

## Conclusions

In conclusion, it is now evident that cardiac exercise rehabilitation can improve the cardiac ejection capacity and myocardial function of elderly patients with chronic heart failure, as predicted. They can promote the activity, proliferation, and invasion of peripheral blood EPC. They also can promote the expression of eNOS and VEGF by upregulating PI3K/AKT pathway to promote angiogenesis and vascular endothelial function. However, the mechanism of the influence of exercise on the number and function of peripheral blood EPC still needs to be further explored.

## Supplementary Information

**Additional file 1: TableS1.** Comparison of heart function indexes between twogroups $\bar{x}$ ± *s* . **Table S2**. Comparisonof serum BNP levels between the two groups. **Table S3**. Comparisonof PI3K, AKT, eNOS, and VEGF mRNA in each group.

## Data Availability

The datasets generated and/or analyzed during the current study are not publicly available but are available from the corresponding author on reasonable request.

## Abbreviations

EPC: Endothelial progenitor cells
BNP: B-natriuretic peptide
MetS: Metabolic equivalents
LVEF: Left ventricular ejection fraction
LVFS: Left ventricular short-axis shortening rate
LVEDD: Left ventricular end-diastolic diameter
LVESD: Left ventricular end-systolic diameter
ELISA: Enzyme-linked immunosorbent assay
