# Supplementary material for: The impact of cardiomotor rehabilitation on endothelial function in elderly patients with chronic heart failure
Source: BMC Cardiovasc Disord. 2021 Nov 1;21:524. doi: 10.1186/s12872-021-02327-5 (PMC8561974; doi:10.1186/s12872-021-02327-5)
Supplement: Supplementary file 1 — Additional file 1: TableS1. Comparison of heart function indexes between twogroups $\bar{x}$x¯ ± s . Table S2. Comparisonof serum BNP levels between the two groups. Table S3. Comparisonof PI3K, AKT, eNOS, and VEGF mRNA in each group. [file 12872_2021_2327_MOESM1_ESM.doc]

**The impact of cardiomotor rehabilitation on endothelial function in elderly patients with chronic heart failure**

Running title: Cardiomotor rehabilitation in elderly CHF patients

Juming Chen^1^, Shenhong Gu^1*^, Yanling Song^1^, Xinbo Ji^1^, Wangyuan Zeng^1^, Xiaoxi Wang^1^, Yachun Wang^1^, Qingfeng Feng^2^

^1^Department of General Practice, The First Affiliated Hospital of Hainan Medical University, Haikou City, Hainan Province, 570102, China

^2^Graduate School, Hainan Medical University, China Medical University, Haikou City, Hainan Province, 571199, China

**^*^Corresponding author:**

Shenhong Gu

Department of General Practice, The First Affiliated Hospital of Hainan Medical University, Haikou City, Hainan Province, 570102, China

E-mail: jill789789@163.com

**Table S1.** Comparison of heart function indexes between two groups ($\bar{x}\pm s$).

| Group | N | LVEF (%) | | | | LVFS (%) | | | |
| --- | --- | --- | --- | --- | --- | --- | --- | --- | --- |
|  |  | Before treatment | After treatment | t | P | Before treatment | After treatment | t | P |
| Control Group | 40 | 43.41 ± 3.28 | 47.97 ± 3.14 | 5.602 | 0.000 | 15.44 ± 3.15 | 18.53 ± 4.07 | 5.897 | 0.000 |
| Exercise rehabilitation group | 40 | 44.73 ± 3.02 | 51.27 ± 3.26 | 7.468 | 0.000 | 15.16 ± 3.08 | 21.89 ± 4.54 | 8.687 | 0.000 |
| t |  | 0.684 | 5.489 |  |  | 0.318 | 5.471 |  |  |
| P |  | 0.433 | 0.000 |  |  | 0.795 | 0.000 |  |  |
| Group | N | LVEDD (mm) | | | | LVESD (mm) | | | |
|  |  | Before treatment | After treatment | t | P | Before treatment | After treatment | t | P |
| Control Group | 40 | 57.38 ± 4.21 | 53.85 ± 4.19 | 5.587 | 0.000 | 48.53 ± 4.24 | 45.12 ± 3.27 | 5.997 | 0.000 |
| Exercise rehabilitation group | 40 | 56.85 ± 3.98 | 50.75 ± 4.16 | 7.321 | 0.000 | 47.38 ± 4.33 | 41.69 ± 3.53 | 8.025 | 0.000 |
| t |  | 0.432 | 5.682 |  |  | 0.384 | 6.187 |  |  |
| P |  | 0.682 | 0.000 |  |  | 0.725 | 0.000 |  |  |

**Table S2.** Comparison of serum BNP levels between the two groups.

| Group | N | Before treatment | After treatment | t | P |
| --- | --- | --- | --- | --- | --- |
| Control Group | 40 | 206.36 ± 24.68 | 174.28 ± 20.86 | 5.449 | 0.000 |
| Exercise rehabilitation group | 40 | 211.72 ± 24.08 | 148.58 ± 21.82 | 7.314 | 0.000 |
| t |  | 0.682 | 5.841 |  |  |
| P |  | 0.312 | 0.000 |  |  |

**Table S3.** Comparison of PI3K, AKT, eNOS and VEGF mRNA in each group.

| Group | PI3K mRNA | AKT mRNA | eNOS mRNA | VEGF mRNA |
| --- | --- | --- | --- | --- |
| Control Group | 2.55 ± 0.57 | 3.04 ± 0.60 | 2.83 ± 0.44 | 2.41 ± 0.46 |
| Exercise rehabilitation group | 4.28 ± 0.98 | 5.16 ± 0.95 | 4.58 ± 0.86 | 5.32 ± 1.05 |
| t | 11.642 | 9.687 | 9.114 | 14.682 |
| P | 0.000 | 0.000 | 0.000 | 0.000 |
